# *Scedosporium apiospermum, Scedosporium aurantiacum, Scedosporium minutisporum* and *Lomentospora prolificans*: a comparative study of surface molecules produced by conidial and germinated conidial cells

**DOI:** 10.1590/0074-02760180102

**Published:** 2018-06-18

**Authors:** Thaís Pereira de Mello, Ana Carolina Aor, Diego de Souza Gonçalves, Sergio Henrique Seabra, Marta Helena Branquinha, André Luis Souza dos Santos

**Affiliations:** 1 Universidade Federal do Rio de Janeiro, Instituto de Microbiologia Paulo de Góes, Departamento de Microbiologia Geral, Laboratório de Estudos Avançados em Microrganismos Emergentes e Resistentes, Rio de Janeiro, RJ, Brasil; 2 Universidade Federal do Rio de Janeiro, Instituto de Química, Programa de Pós-Graduação em Bioquímica, Rio de Janeiro, RJ, Brasil; 3 Centro Universitário Estadual da Zona Oeste, Laboratório de Tecnologia em Cultura de Células, Rio de Janeiro, RJ, Brasil

**Keywords:** Scedosporium/Lomentospora, germination, cell surface, glycoconjugates, polysaccharides, polypeptides

## Abstract

**BACKGROUND:**

*Scedosporium/Lomentospora* species are opportunistic mould pathogens, presenting notable antifungal resistance.

**OBJECTIVES/METHODS:**

We analysed the conidia and germinated conidia of *S. apiospermum* (*Sap*), *S. aurantiacum* (*Sau*), *S. minutisporum* (*Smi*) and *L. prolificans* (*Lpr*) by scanning electron microscopy and exposition of surface molecules by fluorescence microscopy.

**FINDINGS:**

Conidia of *Sap*, *Smi* and *Sau* had oval, ellipsoidal and cylindrical shape, respectively, with several irregularities surrounding all surface areas, whereas *Lpr* conidia were rounded with a smooth surface. The germination of *Sap* occurred at the conidial bottom, while *Smi* and *Sau* germination primarily occurred at the centre of the conidial cell, and *Lpr* germination initiated at any part of the conidial surface. The staining of *N*-acetylglucosamine-containing molecules by fluorescein-labelled WGA primarily occurred during the germination of all studied fungi and in the conidial scars, which is the primary location of germination. Calcofluor white, which recognises the polysaccharide chitin, strongly stained the conidial cells and, to a lesser extent, the germination. Both mannose-rich glycoconjugates (evidenced by fluoresceinated-ConA) and cell wall externally located polypeptides presented distinct surface locations and expression according to both morphotypes and fungal species. In contrast, sialic acid and galactose-containing structures were not detected at fungal surfaces.

**MAIN CONCLUSIONS:**

The present study demonstrated the differential production/exposition of surface molecules on distinct morphotypes of *Scedosporium*/*Lomentospora* species.

*Scedosporium* and *Lomentospora* are saprophytic filamentous fungi found in different human-impacted environments, and the species belonging to these genera have globally emerged as aetiologic agents of localised and disseminated diseases in both immunocompromised and immunocompetent individuals (Cortez et al. 2008). In this context, eumycetoma is considered the main clinical manifestation caused by *Scedosporium*/*Lomentospora* species in healthy persons, while invasive infections are commonly reported, for example, in transplant recipients and patients with haematological diseases. Consistently, *Scedosporium* is the second most frequent filamentous fungal genus (after *Aspergillus*) to colonise the lungs of patients with cystic fibrosis, which can induce a progressive and severe deterioration of the lung function of these individuals over time (Cortez et al. 2008).

*Scedosporium apiospermum*, *Lomentospora prolificans* (formerly *Scedosporium prolificans*) and *Scedosporium aurantiacum* are the most medically important species, which also present typical multidrug-resistance profiles (Lackner and Guarro 2013), while *S. minutisporum* is mainly an environmental species (Gilgado et al. 2009). In all these fungi, infection begins with conidial adherence, for example, into the lungs or subcutaneous tissues, followed by germination and full hyphal development, which can culminate in fungal dissemination through the tissues and/or bloodstream to virtually all organs in the human body (Osherov and May 2001).

Conidial germination is an essential and physiological step during the colonisation of new environments by filamentous fungi, considering both saprophytic and pathogenic growth lifestyles. Moreover, the germination event is the initial step to biofilm formation (Harding et al. 2009). Considering the biological relevance of the germination process, we previously reported that the conidia-into-hyphae differentiation in *S. apiospermum*, *S. minutisporum*, *S. aurantiacum* and *L. prolificans* occurred in a time-dependent manner, with more than 75% of the conidial population germinated after 4 h of incubation on Sabouraud medium under optimum *in vitro* conditions (pH 7.0, 37°C, 5% CO_2_). In addition, similar rates of germination were detected when conidia were incubated in different culture media and at varying pH values; however, the germination rate significantly decreased in all studied fungal species when incubated at the atmospheric level of CO_2_ (0.033%) compared with the CO_2_ concentration found in mammalian tissues. Interestingly, the hyphae were more resistant to itraconazole, fluconazole and voriconazole than the conidial cells (de Mello et al. 2016a). The prolonged incubation of *S. apiospermum*, *S. minutisporum*, *S. aurantiacum* and *L. prolificans* conidia for 24, 48 and 72 h resulted in the formation of classical biofilms, which were characterised by intertwined mycelia connected by an extracellular matrix and enhanced antifungal resistance profile (de Mello et al. 2016b).

The initial interaction between fungi and host structures is mediated by fungal cell surface molecules, including different types of glycoconjugates, such as polysaccharides, (glyco)proteins and (glycol)lipids (Ghamrawi et al. 2014). In this context, until recently, only a limited number of cell surface constituents in *Scedosporium*/*Lomentospora* species have been identified, including (i) peptidorhamnomannans (PRMs), which participate in the adhesion of conidia to epithelial cells as well as in the induction of TNF-α and IL-10 production by macrophages (Pinto et al. 2004, Pinto et al. 2005, Figueiredo et al. 2010, Lopes et al. 2010, Barreto-Bergter et al. 2011, Xisto et al. 2015), (ii) α-glucan, which is involved in the phagocytic process and induces the secretion of TNF-α and IL-12 by macrophages (Bittencourt et al. 2006), (iii) melanin, which masks surface glycoconjugates and affects the conidial surface electrostatic charge (Ghamrawi et al. 2014), and (iv) ceramide monohexosides (CMHs), which are glycolipids involved in fungal differentiation (Pinto et al. 2002, Rollin-Pinheiro et al. 2014).

The conidial germination process is accompanied by multiple biochemical and morphological changes (Osherov and May 2001). Therefore, in the present study, we used fluorescence microscopic approaches to investigate the differential expression of surface-exposed molecules in *S. apiospermum*, *S. minutisporum*, *S. aurantiacum* and *L. prolificans*, considering both conidial and germinated conidial cells. Furthermore, we analysed the surface ultrastructural aspects of these two fungal morphotypes by using scanning electron microscopy.

## MATERIALS AND METHODS

*Chemicals* - Calcofluor white (Cw), fluorescein isothiocyanate (FITC) wheat germ agglutinin (WGA), FITC-Concanavalin A (Con A), biotin-labelled *Limax flavus* agglutinin (LFA), biotin-*Sambucus nigra* agglutinin (SNA), biotin-*Maackia amurensis* agglutinin (MAA), biotin-peanut agglutinin (PNA), 5/6-carboxy-tetramethyl-rhodamine succinimidyl ester (NHS-rhodamine), FITC-streptavidin, *N*-propyl gallate, paraformaldehyde and osmium tetroxide were purchased from Sigma-Aldrich (St Louis, MO, USA). The components of culture media and buffers were obtained from Merck (Darmstadt, Germany). All other reagents were of analytical grade.

*Microorganisms and growth conditions* - *S. apiospermum* HLPB strain was provided by Dr Bodo Wanke (Hospital Evandro Chagas, Instituto Oswaldo Cruz, Rio de Janeiro, Brazil), *L. prolificans* FMR 3569 was provided by Dr Josep Guarro (Facultad de Medicina y Ciencias de la Salud, Reus, Spain), and *S. minutisporum* IHEM21148 and *S. aurantiacum* IHEM21147 were provided by Pr Jean-Philippe Bouchara (Université d'Angers, Angers, France). The fungi were maintained on Sabouraud (2% glucose, 1% peptone, and 0.5% yeast extract) liquid culture medium at ambient temperature with orbital shaking. The conidia were grown at room temperature on Petri dishes containing potato dextrose agar medium (PDA; Difco Laboratories, Detroit, EUA). After seven days in culture, conidial cells were obtained by washing the plate surface with phosphate-buffered saline (PBS; 10 mM NaH_2_PO_4_, 10 mM Na_2_HPO_4_, and 150 mM NaCl; pH 7.2), followed by filtration through a 40 μm nylon cell strainer (BD Falcon, EUA) to remove hyphal fragments (Hohl et al. 2005, de Mello et al. 2016a). The number of conidial cells was counted in a Neubauer chamber.

*Scanning electron microscopy (SEM) analysis* - Conidia and germinated conidia (10^6^ cells) were fixed overnight in a solution containing 2.5% glutaraldehyde in 0.1 M sodium cacodylate buffer (pH 7.2) at 4°C and then washed three times with PBS and fixed with 2% osmium tetroxide for 2 h. The samples were dehydrated in graded concentrations (25-100%) of acetone. The cells were dried by critical point method, mounted on stubs, coated with gold (20-30 nm) and observed under a JEOL JSM 6490LV scanning electron microscope (GenTech Scientific Inc., Arcade, NY, USA) (de Mello et al. 2016b).

*Fluorescence microscopy assays* - Conidia and germinated conidia (10^6^ cells) were fixed for 1 h at room temperature in 0.4% paraformaldehyde diluted in PBS, followed by extensive washing in the same buffer. The fixed cells maintained their morphological integrity, as verified by microscopic observation. The fungal cells were then washed three times in PBS and incubated for 1 h at room temperature with the following markers: Cw at 5 μg/mL, FITC-WGA at 5 μg/mL, FITC-Con A at 25 μg/mL, biotin-PNA at 50 μg/mL, biotin-LFA at 50 μg/mL, biotin-MAA at 50 μg/mL, biotin-SNA at 50 μg/mL and *NHS*-rhodamine at 40 μg/mL. Subsequently, the fungal cells were washed three times with cold PBS. Cells stained with biotin-labelled agglutinins were subsequently incubated with FITC-streptavidin (at 1:200 dilution) for an additional 1 h at room temperature, followed by three washes with PBS. The control systems were composed of fungal cells not incubated with fluorescent markers. To visualise the fungal staining pattern, 10 μL of each system was applied to a microscope slide containing *N*-propyl gallate diluted in PBS:glycerol (1:1, vol/vol). The samples were observed under a fluorescence microscope (Axioplan 2) by using a 63× magnification lens. The images were digitally recorded by using a cooled CCD camera (Color View XS, Analysis GmBH, DE), and analysed by using ANALYSIS system software (AnalySIS, DE). WGA recognises *N*-acetylglucosamine oligomers, Cw binds to the polysaccharide chitin, Con A has affinity for both α-*d*-mannosyl and α-*d*-glucosyl residues, PNA recognises galactose-β(1-3)-*N*-acetylgalactosamine, *NHS*-rhodamine recognises the amino groups from surface-exposed polypeptides, and SNA and MAA specifically recognise α2,6-sialylgalactosyl and α2,3-sialylgalactosyl residues, respectively, while LFA recognises *N*-acetylneuraminic acid residues in any linkage. The percentage of either fluorescent conidial or germinated conidial cells, considering each of the abovementioned markers, was calculated after randomly evaluating 50 fungi in each triplicate. Considering the germinated conidia, the fluorescence of both the conidium body and germination projection was separately evaluated as previously proposed (de Mello et al. 2016a).

*Statistics* - All experiments were performed in triplicate in three independent experimental sets. In all sets of experiments, 50 conidia and 50 germinated conidia were evaluated, and representative images of each system were shown.

## RESULTS AND DISCUSSION

Cellular differentiation is a universal process in which living cells alter their behaviour, including modifications of morphology and modulations of both biochemical and genetic programmes, to adapt to the environmental changes that enable the survival, dissemination and perpetuation of the species. The conidia-into-hyphae differentiation is a crucial event in filamentous fungi but poorly explored in *Scedosporium*/*Lomentospora* species. Thus, we examined this biological event at an ultrastructural perspective as well as by the expression of surface molecules in these fungi.

**Fig. 1:**
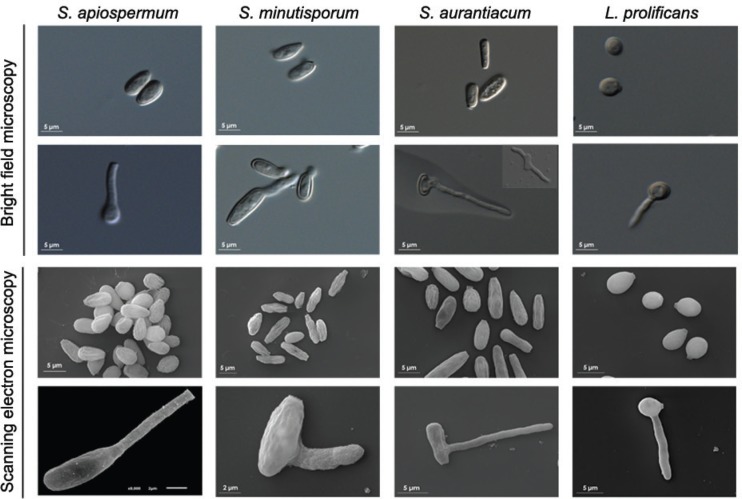
morphology of *Scedosporium apiospermum*, *S. minutisporum*, *S. aurantiacum* and *Lomentospora prolificans*. The conidia and germinated conidia were inspected by both bright field microscopy and scanning electron microscopy.

The conidia of *S. apiospermum* were oval, and the surface has several irregularities and invaginations (Fig. 1); the germination projections were observed only from the bottom of conidia and had smooth surfaces (Fig. 1). The conidia of *S. minutisporum* had an ellipsoidal shape, with one straight edge and a surface with invaginations (Fig. 1); germination begins in the centre of conidial cell, which exhibited a rough surface (Fig. 1) and is similar to the germination surface of *Aspergillus fumigatus* (Rohde et al. 2001). The conidia of *S. aurantiacum* were cylindrical and had invaginations on the surface; germination exhibited a smooth surface and was observed in the centre (Fig. 1) or on both ends of the conidial cell (Fig. 1, inset), enabling better space exploration (de Mello et al. 2016a). Conidia of *L. prolificans* had oval or rounded shapes, and germination was initiated in any part of its surface; both morphotypes had a smooth surface (Fig. 1). This smooth surface is also observed on accessory conidia of *Aspergillus terreus*, whereas phialidic conidium had a striated surface (Deak et al. 2011). In *S. boydii* (Ghamrawi et al. 2015), as observed for *S. minutisporum* and *S. aurantiacum*, germination primarily occurs at the centre of conidia, and not at the conidial bottom, as observed in *S. apiospermum* (in the present study and Stepanova et al. 2016).

The fungal cell wall contains molecules involved in morphogenesis and cell-cell, cell-extracellular matrix and cell-environment interactions (Latgé and Beauvais 2014). Furthermore, the composition of the cell wall can vary according to the cellular morphotype (Beauvais et al. 2014). For example, the outmost layer of *A. fumigatus* conidia is composed of hydrophobin rodlet and melanin, while germinated conidia possess α-1,3-glucans, galactomannans, galactosaminogalactans and *N*-glycosylated proteins on their outer surfaces (Gow et al. 2017). In *Scedosporium* species, only one surface-located molecule was reported, presenting differential expression regarding the distinct fungal morphotypes. In *S. boydii*, the CMH was found on the mycelium surface but not on the conidia surface by immunofluorescence microscopy using an anti-CMH antibody (Pinto et al. 2002). In *S. apiospermum*, the anti-CMH monoclonal antibody strongly recognises molecules on the conidial cell surface and weakly recognises molecules on the mycelial cell surface (Rollin-Pinheiro et al. 2014). To detect differences in the distribution of surface molecules between conidia and germinated conidia, we performed fluorescence microscopy by using different markers to detect glycoconjugates, polysaccharides and polypeptides in *S. apiospermum*, *S. minutisporum*, *S. aurantiacum* and *L. prolificans*.

**Fig. 2:**
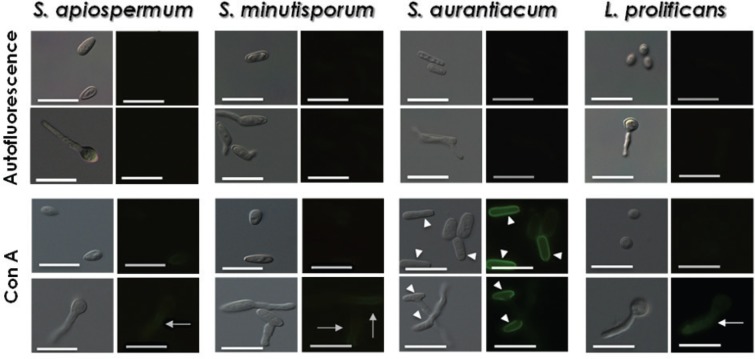
binding of concanavalin A (Con A) to the surface of both conidia and germinated conidia of *Scedosporium apiospermum*, *S. minutisporum*, *S. aurantiacum* and *Lomentospora prolificans*, evidencing the mannose-/glucose-rich glycoconjugates. Experimental systems were analysed under differential interferential contrast images and fluorescence. Control, untreated cells were analysed for autofluorescence. The arrowheads show the prominent labelling in conidial cells, while the arrows show the labelling along all the germination extension. Scale bars = 25 μm.

Regarding the binding of FITC-Con A, 92.9 ± 10.1%, 51.4 ± 12.1% and 4.0 ± 2.8% of conidia from *S. apiospermum*, *S. minutisporum* and *L. prolificans*, respectively, were weakly stained (Table I). Interestingly, after the induction of *in vitro* differentiation, 100% of the fungal cells of those species were stained by Con A, with greater fluorescence intensity on the germination extension compared with the conidial cell (Table I, Fig. 2). In *S. aurantiacum*, 100% of conidia were stained with FITC-Con A; however, two distinct populations were clearly identified with different affinities for this lectin, showing either intense or weak staining (Table I, Fig. 2). A simple explanation for this observation could be that the lack of equal expression is correlated with the *S. aurantiacum* growth phase, since conidial cultures were not synchronised. In addition, the occurrence of distinct subpopulations with differential reactivity with probes for Con A detection could alternatively denote differences in the expression of surface mannose/glucose-rich glycoconjugates or even diminished accessibility to external ligands in cell subsets. On the germinated conidia of *S. aurantiacum*, the labelling is more evident in conidial part than along the length of germination. Consistent with the present findings, Ghamrawi et al. (2014) showed that the conidial population of *S. boydii* was heterogeneously labelled by Con A due to the different ageing times of each conidium. Additionally, these authors demonstrated a progressive increase in the amount of melanin during the maturation of conidia and that this molecule masked the glycoconjugates presented on the fungal surface, which reduced the accessibility to those components by lectin. These hypotheses could also explain why not all *S. apiospermum*, *S. minutisporum* and *L. prolificans* conidia were recognised by Con A. Another interesting finding was the fact that a higher concentration of mannose and/or glucose residues was found in the extension of hyphae. This biological phenomenon could be explained, at least in part, by the increased adhesive properties of fungal hyphae, since mannose-rich glycoconjugates directly participate in the adhesive events of fungal cells to the host structures (e.g., cells, tissues and extracellular matrices) (Osherov and May 2001, Braga-Silva and Santos 2011).

**TABLE I:** Percentage of labeled fungal cells by each surface marker analysed

	*Scedosporium apiospermum*			*Scedosporium minutisporum*			*Scedosporium aurantiacum*			*Lomentospora prolificans*		
Tools	Conidia	Germinated conidia		Conidia	Germinated conidia		Conidia	Germinated conidia		Conidia	Germinated conidia	
		Conidium	Germination		Conidium	Germination		Conidium	Germination		Conidium	Germination
Cw	95.6 ± 6.1	100	100	98.8 ± 1.9	100	100	99 ± 1.6	100	100	100	100	100
WGA	44.2 ± 20.0	21.4 ± 2.0	100	80.0 ± 28.3	25.5 ± 5.0	100	8.8 ± 1.6	0	100	15.5 ± 6.2	0	100
Con A	92.9 ± 10.1	100	100	51.4 ± 12.1	13.3 ± 3.0	100	100	100	100	4.0 ± 2.8	100	100
*NHS*-Rho	96.6 ± 5.8	100	100	98.0 ± 2.8	100	100	82.7 ± 18.0	100	100	92.8 ± 10.0	100	100

Cw: calcofluor white; WGA: white germ agglutinin; Con A: concanavalin A; *NHS*-Rho: 5/6-carboxy-tetramethyl-rhodamine succinimidyl ester.

**Fig. 3:**
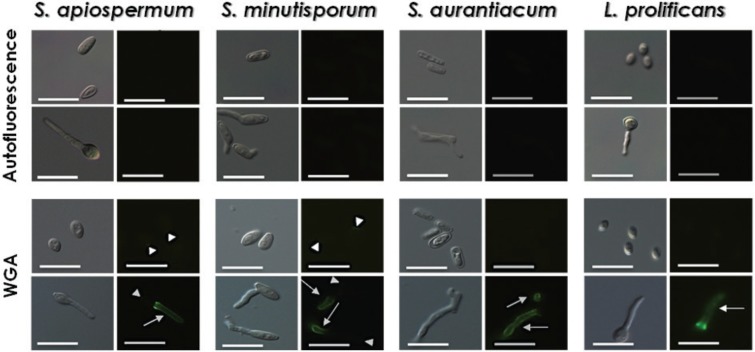
binding of white germ agglutinin (WGA) to the surface of both conidia and germinated conidia of *Scedosporium apiospermum*, *S. minutisporum*, *S. aurantiacum* and *Lomentospora prolificans*, evidencing the *N*-acetylglucosamine residues. Experimental systems were analysed under differential interferential contrast images and fluorescence. Control, untreated cells were analysed for autofluorescence. The arrowheads show the release scar in conidial cells, while the arrows show the labelling along all the germination extension. Scale bars = 25 μm.

WGA specifically bound to the release scar on a conidial basis in *S. minutisporum* (80.0 ± 28.3%), *S. apiospermum* (44.2 ± 20.0%), *L. prolificans* (15.5 ± 6.2%) and *S. aurantiacum* (8.8 ± 1.6%) (Table I, Fig. 3). However, in the germinated conidia of those species, labelling with WGA occurred along the entire length of germination (Fig. 3) in all observed cells (Table I). Similarly, Ghamrawi et al. (2014), Ghamrawi et al. (2015) observed the binding of WGA just on the basis of the conidium of *S. boydii*, a region where releases scar is present, and along the whole germination extension. Since the polymerisation of *N*-acetylglucosamine forms the polysaccharide chitin, we hypothesised that the labelling in the full length of germination is due to the chitin synthesis occurring at the site, as a result of apical cell growth.

**Fig. 4:**
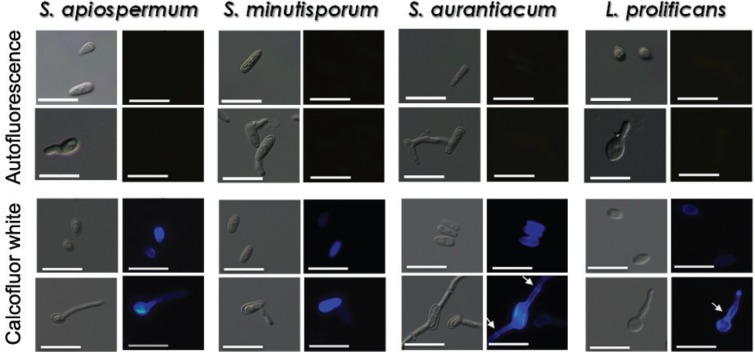
binding of calcofluor white (Cw) to the surface of both conidia and germinated conidia of *Scedosporium apiospermum*, *S. minutisporum*, *S. aurantiacum* and *Lomentospora prolificans*, evidencing the polysaccharide chitin. Experimental systems were analysed under differential interferential contrast images and fluorescence. Control, untreated cells were analysed for autofluorescence. Note the labelling of the septum in the germination (arrows) in *L. prolificans* and *S. aurantiacum*. Scale bars = 25 μm.

Chitin is an essential polysaccharide that forms the main skeleton of the fungal cell wall (Lenardon et al. 2010). Several mechanisms enable the positioning of chitin at specific locations during the cell cycle and under environmental stress conditions (Pellón et al. 2017). For instance, mutants of *A. fumigatus* presenting lower surface chitin concentration were sensitive to adverse environmental conditions, exhibited a higher intracellular osmotic pressure and several abnormalities in morphology, such as hyper-branching hyphae, compared to the parental strain (Masuoka 2004, Muszkieta et al. 2014). To identify potential differences in chitin distribution in conidia and germinated conidia, both morphotypes were stained with Cw. The labelling with Cw in conidia of all species was homogeneous along the entire surface, with some cells more intensely staining than others in the same population (Fig. 4). In germinated conidia of *S. apiospermum* and *S. minutisporum*, the Cw staining was most prominent in the conidia compared with the germination extension. In *L. prolificans* and *S. aurantiacum* germinated conidia, the chitin staining was observed on the entire surface, presenting a more intense fluorescence in the septum (Fig. 4). Previous studies have also demonstrated a higher concentration of chitin in the cell wall of conidia/yeasts than on hyphae in *Candida albicans*, *Histoplasma capsulatum* and *Blastomyces dermattidis* (Domer 1971, Braun and Calderone 1978).

**Fig. 5:**
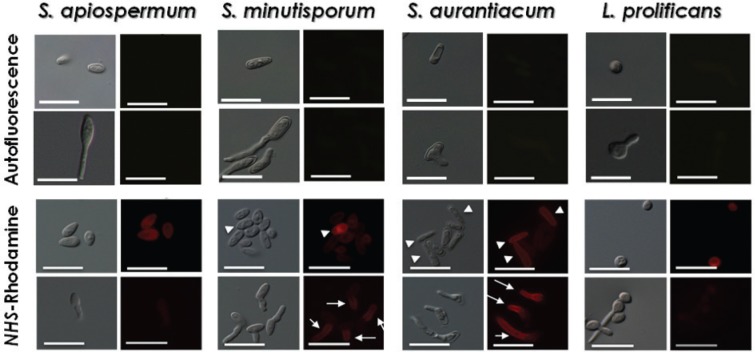
binding of *NHS*-rhodamine to the surface of both conidia and germinated conidia of *Scedosporium apiospermum*, *S. minutisporum*, *S. aurantiacum* and *Lomentospora prolificans*, evidencing the polypeptides. Experimental systems were analysed under differential interferential contrast images and fluorescence. Control, untreated cells were analysed for autofluorescence. The arrowheads show the prominent labelling in conidial cells, while the arrows show the labelling along all the germination extension. Scale bars = 25 μm.

Surface (glyco)proteins are involved in many physiological roles in all living cells. Fungal surface (glycol)proteins, presenting adhesive properties and enzymatic activities, permit cell wall remodelling during conidial germination, adhesion to biotic and abiotic substrates and the cleavage of several host functional structures (Kiffer-Moreira et al. 2007, Ghamrawi et al. 2015). In the present study, we incubated the conidia and germinated conidia of *S. apiospermum*, *S. minutisporum*, *S. aurantiacum* and *L. prolificans* with *NHS*-rhodamine to evaluate cell wall-located polypeptide distribution by fluorescence microscopy. The *NHS*-rhodamine labelling profiles in conidia and germinated conidia of *S. apiospermum* showed no significant differences, showing low fluorescence levels in these two morphotypes (Fig. 5). In *L. prolificans*, the surface polypeptide labelling was more intense in conidia than in germinated conidia (Fig. 5). The percentages of conidia labelled with *NHS*-rhodamine in *S. minutisporum* and *S. aurantiacum* were 98.0 ± 2.8% and 82.7 ± 18.0%, respectively. In germinated conidia of both *S. minutisporum* and *S. aurantiacum*, strong staining was observed on the entire germination extension compared to that in the conidia (Fig. 5). The heterogeneity of the polypeptide labelling in *S. minutisporum* and *S. aurantiacum* conidia could be due to the conidial maturation time, as previously mentioned. Ghamrawi et al. (2015) identified a total of 20 surface glycosylphosphatidylinositol (GPI)-anchored proteins, of which seven of these proteins were detected in both conidia and germinated conidia, while only 12 GPI-anchored proteins were detected in germinated conidia, and only 1 GPI-anchored protein was detected in conidia, demonstrating that not only the concentration of proteins but also the protein type changes during germination.

The presence of galactose-β-(1-3)-*N*-acetylgalactosamine in conidia and germinated conidia of *S. apiospermum*, *S. minutisporum*, *S. aurantiacum* and *L. prolificans* was not detected by fluorescence microscopy by using PNA lectin (data not shown). Similarly, the sialic acid residues were not evidenced on the surfaces of both conidial and germinated conidial cells under the employed experimental conditions by using three distinct sialic acid-recognising lectins (LFA, SNA and MAA) (data not shown). Consistently, sialic acid residues were also not detected on both mycelial and conidial cell surfaces of the dermatophytes *Trichophyton mentagrophytes* and *Trichophyton rubrum* by using different methodologies, including lectins, chemical extraction followed high-performance thin-layer chromatography analysis and colourimetric assays (Esquenazi et al. 2003).

*In conclusion* - Little is known about the conidial-into-hyphae transformation in species belonging to the *Scedosporium* and *Lomentospora* genera. Herein, we demonstrated that the conidia of *S. apiospermum*, *S. minutisporum*, *S. aurantiacum* and *L. prolificans* presented different morphological characteristics, such as size, form, surface sculpturing and local of germination emergence. Moreover, the exposure and/or production of surface molecules (e.g., glycoconjugates, polysaccharides and polypeptides) were evidenced in these human fungal pathogens, showing clear differences in their quantities and surface sites considering either non-germinated or germinated conidial cells (Table II). The differential expression of surface molecules can reflect in the distinct ability of these two morphotypes to colonise distinct environments during their life cycles. How these structures function is still the object of intensive study by our research group. The present results and future studies on the structural characterisation of surface molecules can contribute to the understanding of the molecular changes associated with differentiation of fungal species belonging to *Scedosporium*/*Lomentospora*.

**TABLE II:** Modulation on the expression of surface molecules in *Scedosporium/Lomentospora* spp. along the conidial germination event

	Mannose-rich glycoconjugates (probe: *FITC-Con A*)			*N*-acetylglucosamine-containing molecules (probe: *FITC-WGA*)			Chitin (probe: *calcofluor white*)			Polypeptides (probe: *NHS-rhodamine*)		
Fungal species	Conidia	Germinated conidia		Conidia	Germinated conidia		Conidia	Germinated conidia		Conidia	Germinated conidia	
		Conidium	Germination		Conidium	Germination		Conidium	Germination		Conidium	Germination
*S. apiospermum*												
*S. aurantiacum*												
*S. minutisporum*												
*L. prolificans*												

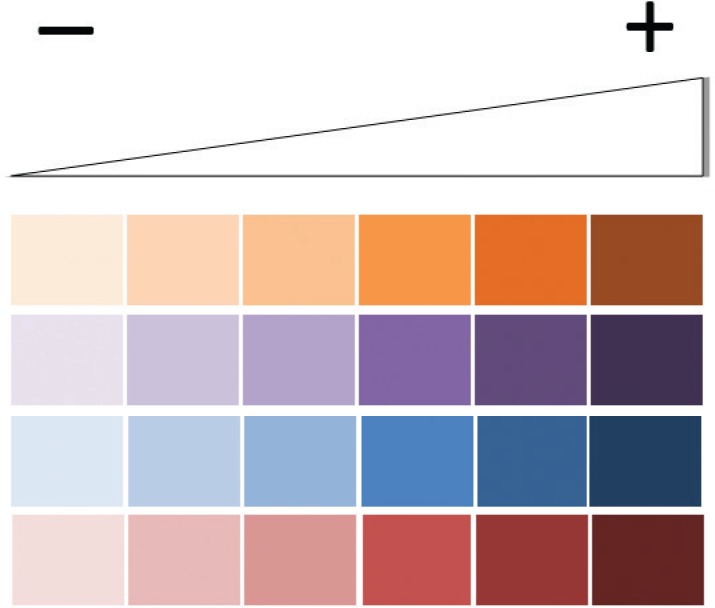

The colors' intensities represent the amount of each studied molecule/structure detected at the surface of *Scedosporium/Lomentospora* spp. as follows: light colors denote lower expression and dark colors denote higher expression.
